# Smartphone addiction and sleep quality in the physical activity-anxiety link: a mediation-moderation model

**DOI:** 10.3389/fpubh.2025.1512812

**Published:** 2025-04-03

**Authors:** Xiangrong Qin, Liangru Liu, Yan Yan, Xuxia Guo, Ningqi Yang, Ling Li

**Affiliations:** School of Physical Education, Guangxi University, Nanning, China

**Keywords:** anxiety, Chinese college students, moderated mediation, physical activity, smartphone addiction, sleep quality

## Abstract

**Background:**

Anxiety symptoms are common among university students in China, posing challenges to mental health. Physical activity may reduce anxiety, but the mechanisms are not fully understood. This study examines how smartphone addiction acts as a mediator and sleep quality as a moderator in the relationship between physical activity and anxiety, aiming to offer theoretical insights and practical strategies for mental health interventions.

**Methods:**

This cross-sectional study was conducted in September 2023 at Guangxi University. A stratified sampling method was used to approach 719 students from diverse physical education classes to distribute questionnaires, and 527 valid questionnaires were returned. Validated instruments included the International Physical Activity Questionnaire-Short Form (IPAQ-SF), Smartphone Addiction Scale-Short Version (SAS-SV), Pittsburgh Sleep Quality Index (PSQI), and Self-Rating Anxiety Scale (SAS). Data analysis involved standardization, descriptive statistics, Pearson correlation, normality testing, mediation and moderation analyses, and Bootstrap validation.

**Results:**

(1) Physical activity was negatively correlated with smartphone addiction (*r* = −0.13, *p* < 0.01). (2) Smartphone addiction was positively correlated with poor sleep quality (*r* = 0.40, *p* < 0.01) and anxiety (*r* = 0.43, *p* < 0.01). (3) Poor sleep quality and anxiety were significantly correlated (*r* = 0.57, *p* < 0.01). (4) A masking effect occurred as the non-significant positive direct effect (*β* = 0.062) was nearly canceled out by the mediation of smartphone addiction (*β* = −0.058), inducing total effect near-zero. (5) Sleep quality significantly influenced the link between smartphone addiction and anxiety, especially in those with poorer sleep, where the impact of smartphone addiction on anxiety was stronger (*β* = 0.061, *p* = 0.036).

**Conclusion:**

This study revealed a more complex relationship between physical activity and anxiety than initially hypothesized. Our findings further revealed the relationship between physical activity and university students’ anxiety, and considered the mediating role of smartphone addiction between the two, as well as the moderating role of sleep quality in the relationship between mobile phone addiction and university students’ anxiety.

## 1 Introduction

In recent years, the world has undergone rapid and continuous developments, presenting both opportunities and challenges for individuals. These significant changes have placed a considerable strain on mental health, particularly among university students (1). In China, university is a crucial time for students as they learn to live independently, handle academic and job pressures, and manage complex social relationships (2). The abrupt and substantial nature of these pressures frequently results in various psychological issues, with anxiety disorders being particularly prevalent (3). Anxiety disorders are typified by enduring and excessive worry, fear, and physiological symptoms that markedly affect emotional regulation, cognitive functions, and daily living (4). Spielberger (5) classified anxiety into two types: state anxiety, a temporary response to specific situations, and trait anxiety, a long-term, persistent condition. Research indicated that consultations related to anxiety are among the most commonly addressed concerns at university mental health centers (6). Ströhle et al. (7) further emphasized that anxiety disorders during university years can adversely affect students’ mental health and may elevate the risk of developing additional neurological disorders in later life. Consequently, it is imperative to develop effective strategies to prevent the onset of anxiety symptoms in college students.

Physical activity is widely advocated for mental health in Chinese students, research shows regular exercise is a powerful intervention for psychological problems (8, 9). Wang et al. (10) found that a 12-week Tai Chi intervention demonstrated significant anxiolytic effects in university students. Furthermore, insufficient physical activity has been recognized as a risk factor for the development of anxiety disorders (11). In a separate study, Mao and Chen (2) study suggested that regular exercise was a protective factor for mental health of Chinese university students. Moreover, a European study of adolescents found that highly active groups had lower anxiety scores than sedentary peers (12). Because all of these studies have shown that physical activity can reduce anxiety. Thus, our first hypothesis posits that there is a negative correlation between physical activity and anxiety among Chinese university students.

The digital era has introduced novel risk pathways, with smartphone addiction emerging as a salient mediator between lifestyle and mental health (13). Specifically, research has identified a notable correlation between the progression of Chinese society and smartphone addiction among university students (14). This was confirmed during the COVID-19 lockdowns, China’s abrupt shift to online education intensified smartphone addiction prevalence (15), simultaneously reducing physical activity engagement and exacerbating anxiety (16, 17). Crucially, a meta-analysis spanning a decade has demonstrated a consistent upward trend in smartphone addiction among Chinese university students from 2013 to 2022 (18), revealed a positive correlation between anxiety disorders and smartphone addiction. Further research has shown that a vicious cycle exists between smartphone addiction and anxiety among Chinese university students, where each condition mutually influences and exacerbates the symptoms of the other (19). However, it is worth noting that emerging studies have demonstrated that physical activity can effectively mitigate smartphone addiction behaviors, which may indirectly contribute to reduced anxiety symptoms (20, 21). Based on these evidences, we hypothesize that smartphone addiction plays a specific mediating role between physical activity and anxiety disorders. Therefore, our second hypothesis posits that smartphone addiction mediates the relationship between physical activity and anxiety among Chinese university students.

Sleep quality may further regulate the mediating role of smartphone addiction through biological and behavioral pathways. Research shows that the sleep quality of university students is significantly affected by smartphone addiction. (22). Yao et al. (23) conducted a cross-lagged panel analysis among 807 Chinese university students, revealing a bidirectional relationship between smartphone addiction and insomnia over time. This harmful cycle shows how smartphone use, especially at night, negatively affects sleep by delaying sleep onset (24) and reducing sleep quality (25). A cross-sectional survey conducted by Matar Boumosleh et al. (26) involving 688 undergraduates found that 38.1% of the students had poor sleep quality due to frequent smartphone use at night. In addition, high-quality sleep is essential for the preservation of mental health, and insufficient sleep can impair an individual’s capacity to manage daily stressors, thereby intensifying symptoms of anxiety (27). Thus, the study conducted by Zhu et al. (28) identified that students with adequate sleep exhibit lower anxiety despite similar smartphone addition levels. Based on these findings, we hypothesize that sleep quality exerts a moderating role in the “smartphone addiction → anxiety” pathway. In 2012, Billieux et al. (29) study provides scientific evidence supporting this speculation: smartphone addiction disrupts nighttime sleep rhythm and increases the risk of anxiety disorders. A recent systematic review further demonstrated the co-occurrence of smartphone addiction, poor sleep quality, and anxiety disorders among college student populations (30), which reinforces our hypothesis. Consequently, our final hypothesis posits that sleep quality may mediate the relationship between smartphone addiction and anxiety among Chinese university students.

**Figure 1:**
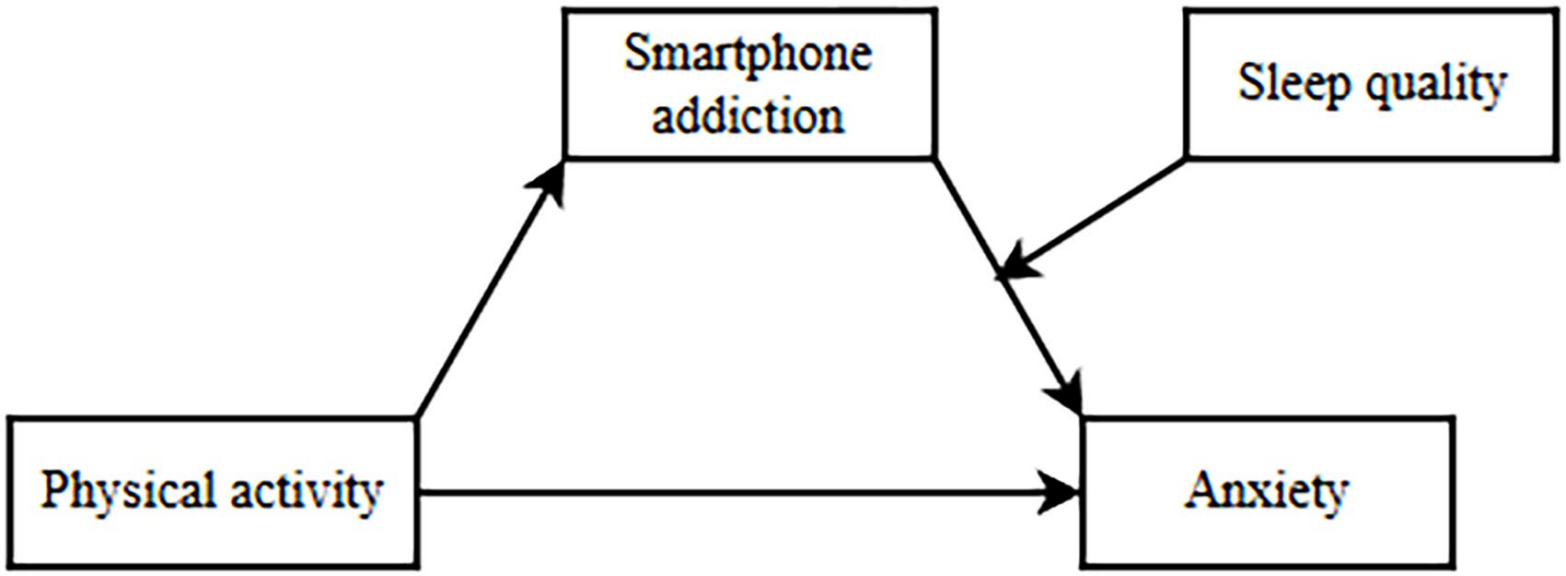
Hypothesized model.

Although existing studies have elucidated the intricate interactions among physical activity, smartphone addiction, and sleep quality, they frequently concentrate on the relationship between a single variable and anxiety, thereby neglecting a comprehensive understanding of the interplay among physical activity, smartphone addiction, and sleep quality. Moreover, the mediating and moderating roles of physical activity in mitigating anxiety have not been thoroughly investigated. This study seeks to develop a model that integrates both mediating and moderating effects to systematically examine the interrelationships among physical activity, smartphone addiction, sleep quality, and anxiety. The proposed hypothesis posits that physical activity (X) may indirectly mitigate anxiety (Y) through its impact on smartphone addiction (M), with the strength of this mediating effect varying according to different levels of sleep quality (W). This research aims to comprehensively investigate the interactions among these variables and their impact on anxiety levels among Chinese university students. The findings are expected to inform the development of targeted intervention strategies to address student anxiety, ultimately contributing to the improvement of mental health within this demographic. A theoretical model diagram is constructed (see Figure 1).

## 2 Methods

### 2.1 Participants and procedure

This cross-sectional study was undertaken in September 2023 at Guangxi University. A stratified random sampling technique was utilized to select participants from various undergraduate public physical education classes across the campus, encompassing a total of 10 classes. The required sample size for the smartphone addiction student population was computed as 255 using Epi Info 7 (version 7.2.6.0) [population size: 23,961 undergraduates; expected prevalence: 21.3% (31); acceptable margin of error: 5% (32); design effect: 1; confidence level: 95%]. The required sample size for anxiety disorder student population of 300 was computed using Epi Info 7 (version 7.2.6.0) [population size: 23,961 undergraduates; expected prevalence: 27.2% (33); acceptable margin of error: 5% (32); design effect: 1; confidence level: 95%]. The population base of 23,961 undergraduate students was obtained from Guangxi University’s official website^1^ during our data collection period. This number encompassed all full-time undergraduates across all four academic years (Years 1–4), and did not include graduate students. The expected prevalence rates of smartphone addiction and anxiety were derived from Long et al. (31) and Mao (33) respective studies, which examined prevalence among Chinese college students. Allowable error was set at 5% following standard epidemiological protocols as used in the study by Nikolic et al. (32). Considering the possibility of incomplete or invalid questionnaires, more students were incorporated into the study.

Data collection was facilitated through the “Wenjuanxing” platform,^2^ resulting in 719 students completing questionnaires that comprised demographic information and pertinent scales, resulted in a 100% response rate. Before distributing the questionnaires, trained researchers conducted a comprehensive briefing for participants, addressing the anonymity, confidentiality, and objectives of the survey, and emphasizing the importance of voluntary participation and informed consent. Informed consent was duly obtained from all participants, thereby upholding the ethical and legal standards of the study. To ensure the quality and reliability of the collected data, stringent screening criteria were implemented. Questionnaires were excluded if they were completed in less than 120 s, contained any missing data, or exhibited anomalous scoring patterns (either excessively high or low). The 120-s threshold was empirically justified through preliminary testing showing even content-familiar researchers required ≥120 s for conscientious completion. This criterion corresponds with Callegaro et al.’s methodology (34) linking rushed responses to data inaccuracy, ensuring integrity through systematic exclusion of inattentive responses while preserving instrument rigor. Following the screening process, 192 questionnaires were excluded, a total of 527 valid questionnaires were collected, resulting in an effective response rate of 73.3%. The study was in accordance with the relevant guidelines and regulations such as the declaration of Helsinki, and the study protocol received approval from the Ethics Review Committee of Guangxi University (No. GXU-2024-066), with informed consent from all participants.

### 2.2 Measures

#### 2.2.1 International Physical Activity Questionnaire—short form

The participants’ physical activity levels were evaluated utilizing the abbreviated version of the International Physical Activity Questionnaire (35). The IPAQ-SF consists of 7 questions, 6 of which ask about individual physical activity levels and 1 about sitting time. In the short form, the MET value assigned to walking is 3.3, to moderate-intensity activity is 4.0, and to high-intensity activity is 8.0 (36). Both the long and short forms of the IPAQ have demonstrated considerable reliability and reasonable validity in studies across multiple countries (37). The total weekly IPAQ-SF MET activity for each participant is calculated as follows (38):

$$\begin{array}{l} \mathrm{Activity}\;\mathrm{MET}\;\mathrm{value}=\left(\mathrm{no}.\mathrm{of}\;\mathrm{days}\right)\times \left(\mathrm{no}.\mathrm{of}\;\mathrm{minutes}\right)\times \\ \left(\mathrm{activity}\;\mathrm{MET}\;\mathrm{value}\right)\text{.} \end{array}$$

#### 2.2.2 Smartphone Addiction Scale—Short Version

The Smartphone Addiction Scale—Short Version (SAS-SV) (39) consists of 10 items rated on a 6-point Likert scale, with total scores ranging from 10 to 60. This instrument is employed to evaluate the degree of smartphone addiction among participants, with elevated scores reflecting a heightened propensity for addiction. According to the criteria established by Kwon (39), the addiction thresholds are delineated at 31 for males and 33 for females. The gender-specific thresholds (male = 31, female = 33) were derived through Korean adolescents in Kwon’s validation study (39). Cross-cultural validation in Chinese undergraduate students (40) confirmed measurement invariance across populations, coupled with excellent reliability. Internal consistency in this sample was good (Cronbach’s *α* = 0.87) (41).

#### 2.2.3 Pittsburgh Sleep Quality Index

The Pittsburgh Sleep Quality Index (PSQI) (42) comprises 19 items that are grouped into seven component scores: sleep quality, sleep latency, sleep duration, habitual sleep efficiency, sleep disturbances, use of sleep medication, and daytime dysfunction. These components collectively evaluate the overall sleep quality of participants. Each component is scored from 0 to 3, with total scores ranging from 0 to 21; higher scores reflect poorer sleep quality. The PSQI has been validated through stratified cluster sampling of Chinese undergraduates (43). Internal consistency in this sample was acceptable (Cronbach’s *α* = 0.68) (41).

#### 2.2.4 Self-Rating Anxiety Scale

The Self-Rating Anxiety Scale (SAS) (44) contains 20 items, with items 5, 9, 13, 17, and 19 scored inversely. This scale uses a 4-point Likert scale. To achieve clinical applicability and standardized management, Zung (44) applied a conversion by multiplying the scores by 1.25 to adjust the total score range from 20 to 80 to 25 to 100 points. The overall measure of participants’ anxiety levels is determined by calculating the sum of scores across all items. Total standard scores range from 25 (indicating low anxiety) to 100 (indicating high anxiety). Xu et al. (45) stated that, according to the Chinese normative standard, a cutoff standard score of 50 differentiates between non-anxious (<50) and anxious (≥50) statuses. Internal consistency in this sample was acceptable (Cronbach’s *α* = 0.83) (41).

### 2.3 Data analysis

In this study, all data analyses were performed utilizing SPSS software version 29.0. Given the notable discrepancies in the scores obtained from various scales, the scores were initially standardized. Subsequently, descriptive statistical analyses were conducted, and the reliability of the scales was evaluated through internal consistency assessments. Furthermore, the interrelationships among variables were examined using Pearson correlation analysis (46). Following an initial verification of the correlations among the variables, Model 4 of Hayes’ PROCESS macro (version 4.2) was employed to investigate the mediating role of smartphone addiction behaviors in the relationship between physical activity levels and anxiety. Given that the normality tests for all variables yielded *p* < 0.01, indicating significant departures from normal distribution, a bias-corrected non-parametric percentile Bootstrap method with 5,000 resamples was utilized to evaluate the significance of the mediation effects (47). If the confidence interval excludes zero, the mediation effect is deemed statistically significant (*p* < 0.05) (48). To verify the robustness of the results, we employed Bootstrap resampling with 10,000 replications. We observed that the BootSE fluctuation between two sampling results from the same dataset was <10%, with a relative deviation of <5%, meeting the robustness criteria for Bootstrap methods (49, 50). Subsequently, a moderated mediation analysis was performed utilizing Model 14 of the PROCESS macro, with statistical significance assessed through the Bootstrap method. A 95% confidence interval that excludes zero signifies statistically significant findings. The entire analysis constructed a model with physical activity level as the independent variable (X), anxiety as the dependent variable (Y), smartphone addiction behavior as the mediating variable (M), and sleep quality as the moderating variable (W).

## 3 Results

### 3.1 Participant’s characteristics

The participants were between 18 and 22 years old, with 178 males (33.8%) and 349 females (66.2%), other demographics were shown in Table 1. The participant’s characteristics did not have a significant effect when included as a covariate in the models.

**Table 1:** Valid sample demographics.

Variable	Options	n	Percentage (%)
Age	18–22 years	527	100%
Gender	Male	178	33.8%
	Female	349	66.2%
Grade	Freshman	207	39.3%
	Sophomore	245	46.5%
	Junior	67	12.7%
	Senior	8	1.5%
Only child	Yes	139	26.4%
	No	388	73.6%
Place of residence	Rural	319	60.5%
	Urban	208	39.5%
Household income	≤3,000¥	96	18.2%
	3,000 ~ 5,000¥	183	34.7%
	5,000 ~ 8,000¥	115	21.8%
	8,000 ~ 10,000¥	61	11.6%
	>10,000¥	72	13.7%

### 3.2 Harman’s single factor test

Data collection for this study was carried out through self-assessment, a method that may introduce certain common methodological biases. To analyze the data, the Harman single-factor test was employed for factor analysis on all items included in the study. The results of the exploratory factor analysis revealed the extraction of 19 factors with eigenvalues exceeding 1. Notably, the first two factors accounted for 21.724 and 8.326% of the explained variance, respectively, resulting in a cumulative variance explanation of 30.05%, which falls significantly short of the critical threshold of 40%. This suggests that the data in this study are not affected by common method bias (51).

### 3.3 Correlation analysis of main indicators

The correlations presented in Table 2, utilizing physical activity, smartphone addiction, anxiety, and sleep quality as variables, reveal several significant relationships. Notably, there is a significant negative correlation between physical activity and smartphone addiction, with a coefficient of −0.13 (*p* < 0.01). Conversely, a significant positive correlation exists between smartphone addiction and sleep quality, evidenced by a coefficient of 0.40 (*p* < 0.01). Furthermore, the data indicate a significant positive correlation between sleep quality and anxiety, with a coefficient of 0.57 (*p* < 0.01). Additionally, the correlation between anxiety and smartphone addiction is significantly positive, with a coefficient of 0.43 (*p* < 0.01).

**Table 2:** Pearson correlation matrix among the variables.

Variant	M ± SD	Physical activity	Smartphone addiction	Sleep quality	Anxiety
Physical activity	3396.98 ± 2998.69	1.00			
Smartphone addiction	5.77 ± 2.94	−0.13^**^	1.00		
Sleep quality	25.40 ± 7.03	−0.04	0.40^**^	1.00	
Anxiety	42.30 ± 9.05	0.00	0.43^**^	0.57^**^	1.00

*p < 0.05, **p < 0.01.

Overall, physical activity exhibits a weaker correlation with other variables, whereas smartphone addiction demonstrates a significant positive correlation with both sleep quality and anxiety. Additionally, there is a significant positive correlation between anxiety and sleep quality. These significant correlations among the primary variables indicate the necessity for further investigation into potential mediating effects (52, 53).

### 3.4 Testing the mediating effects of smartphone addiction

To evaluate the mediating role of smartphone addiction in the relationship between physical activity and anxiety, we designated physical activity as the independent variable (X), anxiety as the dependent variable (Y), and smartphone addiction as the mediator (M). A mediation regression analysis was performed utilizing Model 4 in the PROCESS macro (54). Model 4 is widely used to test mediation effects. We used this model to analyze the mediating role of smartphone addiction behavior in the relationship between physical activity level and anxiety. Using Monte Carlo Power Analysis for Indirect Effects (55), we calculate that the statistical power of Model 4 is 0.84. The findings, as delineated in Table 3, indicate that physical activity significantly and negatively predict smartphone addiction among college students. (*β* = −0.132, SE = 0.043, 95% CI: [−0.217, − 0.047], *p* < 0.01). Moreover, the direct impact of physical activity on anxiety symptoms among college students was found to be non-significant (*β* = 0.062, SE = 0.040, 95% CI: [−0.016, 0.140], *p* = 0.122). However, smartphone addiction emerged as a significant predictor of anxiety. (*β* = 0.438, SE = 0.040, 95% CI: [0.360, 0.516], *p* < 0.01). The analysis of indirect effects indicates that physical activity exerts a significant negative indirect effect on anxiety through the intermediary variable of smartphone addiction (*β* = −0.058, BootSE = 0.021, 95% CI: [−0.101, −0.017]). This finding meets the criteria for testing mediation effects as outlined by Wen et al. (46) and substantiates the mediating role of smartphone addiction in the relationship between physical activity and anxiety.

**Table 3:** Regression analysis of mediating role of smartphone addiction (*N* = 527).

Regression equation		Overall fit index			Significance of regression coefficient		
Outcome variable	Predictive variable	*R*^2^	*F*	*p*	*β*	95%CI	*t*
Anxiety	Physical activity	0.017	9.278	0.002	−0.132	[−0.217, −0.047]	−3.046 **
Smartphone addiction	Physical activity	0.189	60.897	0.000	0.062	[−0.016, 0.140]	1.550
Anxiety	Smartphone addiction	0.189	60.897	0.000	0.438	[0.360, 0.516]	11.036 **

**p < 0.01.

**Figure 2:**
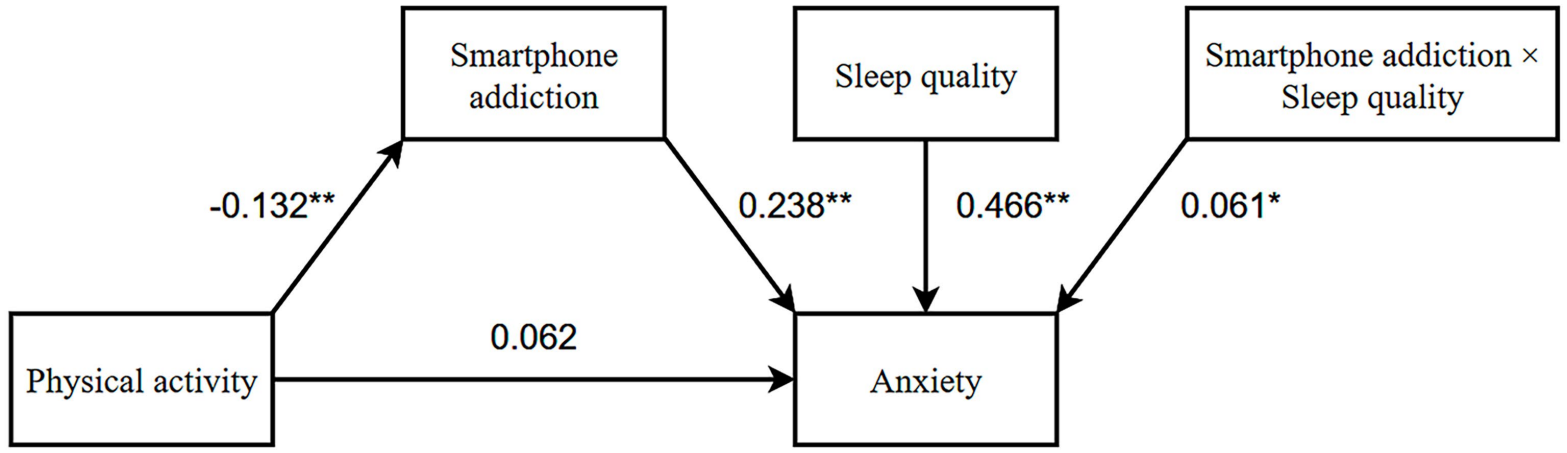
Model of mediating regulation between physical activity and anxiety. *p < 0.05; **p < 0.01.

As demonstrated in Table 4, the direct positive effect of physical activity on anxiety was not statistically significant (*β* = 0.062, 95% CI: [−0.016, 0.140]), while the significant indirect pathway through reduced smartphone addiction (*β* = −0.058, 95% CI: [−0.101, −0.016]) resulted in a near-zero total effect (*β* = 0.004). This pattern meets the key diagnostic criteria for a masking effect, characterized by countervailing direct and indirect pathways operating in opposing directions (56). In our study, it is a phenomenon where the mediator (smartphone addiction) suppresses the true relationship between predictor (physical activity) and outcome (anxiety) (57). This masking explains why the total effect (*β* = 0.004) appears negligible (57): the direct and indirect pathways essentially cancel each other [Total effect = Direct + Indirect: 0.062 + (−0.058) = 0.004)]. These findings underscore the intricate relationship between physical activity and anxiety, suggesting the need for further exploration of additional influencing factors, such as sleep quality, which may serve as a moderating variable in this mediation mechanism (see Figure 2).

**Table 4:** Regression analysis of the mediating role of smartphone addiction (*N* = 527).

Type of effect	Path	Effect size	BootSE	Boot95%CI	
				Lower limit	Upper limit
Indirect effect	Physical activity → Smartphone addiction → Anxiety	−0.058	0.021	−0.101	−0.016
Direct effect	Physical activity →Anxiety	0.062	0.040	−0.016	0.140
Total effect	(Physical activity → Smartphone addiction → Anxiety) + (Physical activity → Anxiety)	0.004	0.044	−0.082	0.090

### 3.5 Testing the moderated mediation effects of smartphone addiction on sleep quality

After confirming the mediating effects of smartphone addiction, we employed the PROCESS macro Model 59 to further investigate the moderating role of sleep quality (25). The moderation by sleep quality was predominantly observed in the latter stages of the mediation model. For a more comprehensive analysis, we utilized PROCESS macro Model 14 (49, 58) to examine the direct and indirect effects of physical activity, smartphone addiction, and sleep quality on depression levels among college students, while controlling for mediating and moderating variables. Using the WebPower R package (59), we calculated the statistical power of Model 14. The power of the conditional indirect effect of physical activity on anxiety through smartphone addition is 0.86, and the power of sleep quality on the path of smartphone addition to anxiety is 0.96.

The analysis (Table 5) indicated that higher levels of physical activity were significantly associated with lower levels of smartphone addiction (*β* = −0.132, SE = 0.043, 95% CI: [−0.217, −0.047], *p* < 0.01). Furthermore, smartphone addiction was found to be a significant positive predictor of anxiety (*β* = 0.238, SE = 0.038, 95% CI: [0.162, 0.313], *p* < 0.01), and sleep quality was also identified as a significant positive predictor of anxiety (*β* = 0.466, SE = 0.038, 95% CI: [0.391, 0.540], *p* < 0.001).

**Table 5:** Regression analysis of sleep quality.

Dependent variable		Overall fit index			Significance of regression coefficients				
Outcome variable	Predictor variable	*R*	*R*^2^	*F*	*β*	SE	LLCI	ULCI	*t*
Anxiety		0.614	0.377	79.090					
	Physical activity				0.052	0.035	−0.016	0.121	1.494
	Smartphone addiction				0.238	0.038	0.162	0.313	6.170**
	Sleep quality				0.466	0.038	0.391	0.540	12.354**
	Product of smartphone addiction and sleep quality				0.061	0.029	0.004	0.118	2.101*

**p < 0.01, *p < 0.05.

Subsequent moderation analysis indicated that sleep quality significantly moderated the indirect effects of smartphone addiction on anxiety (*β* = 0.061, SE = 0.029, 95% CI: [0.004, 0.118], *p* = 0.036). In other words, for each one-unit improvement in sleep quality, the impact of smartphone addiction on anxiety increased by 0.061 units. Specifically, the magnitude of these indirect effects varied considerably across different levels of sleep quality: under conditions of poor sleep quality (high PSQI total score), the indirect effect was −0.040 (SE = 0.016, 95% CI: [−0.073,−0.011], *p* < 0.01); for moderate sleep quality (medium PSQI score), the effect was −0.029 (SE = 0.012, 95% CI: [−0.056,−0.008], *p* < 0.01); and under good sleep quality (low PSQI score), the effect was −0.024 (SE = 0.012, 95% CI: [−0.050,−0.005], *p* < 0.01). These findings indicate that diminished sleep quality substantially intensifies the adverse effects of smartphone addiction on anxiety symptoms, suggesting that the influence of smartphone addiction on anxiety is more pronounced in the context of inadequate sleep.

**Figure 3:**
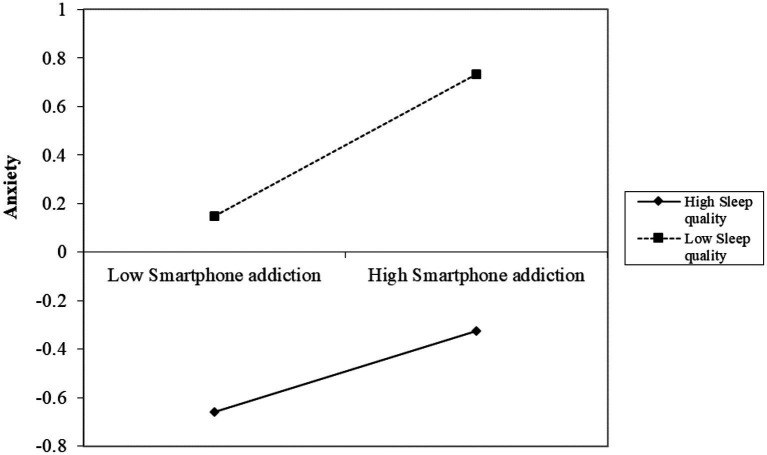
Moderating effect of sleep quality on the relationship between physical activity and anxiety.

To further examine the influence of sleep quality on the relationship between smartphone addiction and anxiety, we plotted simple slopes, as illustrated in Figure 3. The graph indicates a positive trend in the relationship between smartphone addiction and anxiety across varying levels of sleep quality. Notably, when sleep quality is high (*Z* = −0.942), the effect of smartphone addiction on anxiety is significant (*β* = 0.180, SE = 0.051, 95% CI: [0.081, 0.280], *p* < 0.01). As sleep quality declines to a moderate level (*Z* = −0.262), the observed effect intensifies (*β* = 0.222, SE = 0.040, 95% CI: [0.142, 0.301], *p* < 0.01). At poor sleep quality levels (*Z* = 1.097), this effect becomes even more pronounced (*β* = 0.304, SE = 0.046, 95% CI: [0.214, 0.394], *p* < 0.01). These findings indicate that the relationship between smartphone addiction and anxiety is more evident when sleep quality is poor, with all effects reaching statistical significance (*p* < 0.01).

## 4 Discussion

The absence of a significant direct relationship between physical activity and anxiety in our study, contrary to our initial hypothesis: “there is a negative correlation between physical activity and anxiety among Chinese university students.” Mediation analysis demonstrated a significant negative indirect effect through smartphone addiction, indicate that physical activity may indirectly mitigate anxiety by reducing smartphone addiction. This negative profile effect counteracted the positive direct effect, yielding a near-zero total effect. This masked effect indicates that exclusive reliance on total effects may profoundly underestimate the psychological benefits of physical activity, necessitating mediation frameworks to unravel its multidimensional mechanisms. This suggests that our original theoretical model may have oversimplified the complex relationship between physical activity and anxiety. Of particular note, the positive trend in direct effects requires cautious interpretation, even if it is not significant. But it potentially reflects context-specific risks of exercise-induced anxiety, such as, physical fitness assessment pressure (60), athletic performance anxiety (61, 62). Given that our study specifically sampled participants from physical education courses, we posit that evaluation apprehension regarding PE course assessments may have contributed to the observed positive association trend between physical activity and anxiety.

Although constituting a theoretical paradox with our initial hypothesis. However, our findings indicate that smartphone addiction serves as a significant mediating factor between physical activity and anxiety. This supports the perspective of Rodriguez-Ayllon et al. (63), who suggested that the relationship between physical activity and anxiety in preschool children, children, and adolescents may be influenced by potential moderating and mediating factors. Furthermore, our results illustrate that the relationship between physical activity and anxiety is indirect, aligning with the conclusions drawn by Yang et al. (64). From a theoretical standpoint, the findings align with the behavioral substitution theory (65), indicating that engagement in positive physical activity may mitigate anxiety symptoms associated with excessive smartphone use (66). This substitution effect is potentially attributable to the beneficial physiological and psychological responses elicited by physical activity, including elevated endorphin levels (67), improved sleep quality (68), and enhanced self-efficacy (69). For example, studies have proved that physical exercise can effectively improve the self-efficacy of universities students, thereby enhancing their self-confidence and alleviating and inhibiting the expression of anxiety. The substitution effect was statistically demonstrated through a significant indirect pathway wherein physical activity reduced anxiety by mitigating smartphone addiction. This finding empirically validates the displacing hypothesis, wherein adaptive exercise behaviors counteract pathological technology use. In layman’s terms, the time displacement theory suggests that within the fixed 24-h day, increasing time spent on physical activity naturally reduces smartphone usage duration. In China, smartphone addiction is prevalent among university students (70), and this dependency correlates with an increased frequency of social media use, which subsequently exacerbates stress associated with social media engagement (20). For example, heightened social media usage may cause students to excessively scrutinize and compare their body image, resulting in negative self-assessments and elevated psychological stress (71). Furthermore, the internalization of media (72, 73) and the consequent media pressure (74) may intensify the issue of smartphone dependency (75) and exacerbate anxiety symptoms (76). Smartphone addiction can also lead to significant distractions in daily life (77), as students may excessively engage with social media and online interactions at the expense of real-life responsibilities (78). This distracting behavior has the potential to increase psychological and emotional burdens, thereby worsening anxiety (79). A substantial body of research corroborates the positive correlation between smartphone addiction and anxiety among university students (26, 30, 80, 81). In light of these evidences, our study provides robust support for our second hypothesis: smartphone addiction mediates the relationship between physical activity and anxiety among Chinese university students.

It is important to acknowledge the complex bidirectional relationship between smartphone addiction and anxiety. In high-academic-pressure environments, students’ compensatory smartphone usage for escapism or stress regulation may paradoxically amplify anxiety through neurocognitive mechanisms (32). This process establishes a self-perpetuating “stress-addiction-anxiety” triad characterized by. This relationship may fuel a maladaptive feedback loop: anxiety-driven compulsive screen exposure → melatonin suppression and sleep architecture fragmentation → impaired prefrontal cortex functioning (20) → exacerbated anxiety vulnerability. Previous researches suggested that anxiety may also intensify smartphone dependence through various mechanisms (29, 82). Anxious individuals may turn to smartphones as a maladaptive coping strategy, seeking temporary relief through social media, gaming, or other digital activities. This can create a self-reinforcing cycle where anxiety drives increased smartphone use, which in turn may exacerbate anxiety through disrupted sleep patterns, reduced face-to-face social interaction, and increased exposure to anxiety-inducing content (19).

Furthermore, this study elucidates the critical moderating role of sleep quality in the relationship between smartphone addiction and anxiety among Chinese university students. An extensive literature review reveals a complex bidirectional relationship between sleep quality and smartphone addiction. High sleep quality significantly mitigates smartphone dependency by enhancing emotional regulation and stress resilience, thereby diminishing excessive reliance on smartphones (83). Conversely, excessive smartphone usage has the potential to impair sleep quality by disrupting deep sleep and prolonging sleep onset (84–87). This deterioration in sleep quality subsequently exacerbates anxiety levels, thereby creating a detrimental cycle (88). Consequently, a comprehensive understanding and regulation of the interplay between smartphone use and sleep quality may serve as an effective strategy for mitigating anxiety. These findings provide robust support for our final hypothesis: sleep quality may mediate the relationship between smartphone addiction and anxiety among Chinese university students. Based on this, universities should consider incorporating the promotion of good sleep habits as part of their mental health programs. This may include educating students about the importance of sleep and strategies for improving sleep quality, such as setting a curfew for lights out in dormitories. For students experiencing sleep disorders, the university should implement interventions, such as music or meditation, to help alleviate their sleep-related issues (89).

Therefore, educational institutions should fully recognize the significant impact of smartphone addiction on students’ mental health and take proactive measures to address it (32). For example, universities can regularly organize health seminars to raise awareness among students about the psychological risks associated with excessive smartphone use, thereby enhancing their understanding of these issues. Additionally, such seminars can encourage students to consciously reduce their smartphone usage, resist the “digital venting” phenomenon, and offer alternative strategies for stress relief, such as physical activity, social interaction, and mindfulness practices. Furthermore, excessive smartphone use has been shown to impair sleep quality, resulting in insufficient or disrupted sleep, which subsequently affects mental health (90). Given these findings, it is advisable for higher education institutions to prioritize the development of integrated health promotion initiatives. When designing interventions to mitigate student anxiety, considering these multifaceted relationships into program frameworks represents a strategically sound methodology. This could be achieved through the addition of sports facilities, the organization of regular sports events and workshops, and the encouragement of student participation in sports. Such initiatives aim to reduce dependency on smart devices and, consequently, enhance overall mental health. Finally, given the moderating role of sleep quality in the relationship between smartphone addiction and anxiety, future research should investigate how sleep interventions could effectively complement physical activity in mitigating psychological issues associated with technology dependency.

## 5 Implications and limitations

The significance of this study resides in its pioneering investigation into the mediating role of smartphone addiction in the relationship between physical activity and anxiety among Chinese university students. Additionally, the study substantiates the moderating effect of sleep quality on the influence of smartphone addiction on anxiety. Consequently, when examining the relationship between physical activity and anxiety in this demographic, the potential impact of smartphone addiction must be considered. To mitigate issues stemming from smartphone addiction, it is advisable to guide students in adjusting their schedules accordingly.

Although this study provides important insights into the mediating role of smartphone addiction between physical activity and anxiety and the mediating role of sleep quality in this relationship, the following limitations and biases need to be considered when interpreting the findings.

Firstly, we need to acknowledge the flaws of this theoretical model. While the foundational hypothesis was not empirically supported, the non-significant positive trend may reflect anxiety-inducing factors specific to certain subpopulations – particularly physical fitness assessment pressures (60) observed among physical education course enrollees. This suggests two critical limitations in current theoretical frameworks: insufficient incorporation of situational moderators in exercise contexts; oversimplification of physical activity’s unidirectional linear effects on psychological states. Future models should adopt dynamic approaches to delineate boundary conditions underlying physical activity’s dual-edged effects on mental health.

Secondly, the sample is limited to undergraduates from a single university in Guangxi Province, potentially limiting the generalizability of the findings. College students in different regions may differ in their behavior patterns, cultural backgrounds, and mental health status, so the findings may not be broadly applicable to college students in other regions. Therefore, subsequent research endeavors should broaden the sample population to encompass university students from diverse geographical regions, thereby augmenting the generalizability of the findings.

Thirdly, the data in the present study are obtained from self-reports, which may produce self-report response bias. For instance, participants may tend to underestimate negative behaviors, such as smartphone addiction, while overestimating positive behaviors, such as physical activity. Additionally, participants may possess distorted perceptions of their anxiety levels. Although validated scales were employed, the potential for inaccuracies in participants’ self-perception and reporting remains unavoidable. The PSQI scale used in this study presents additional limitations. Its multidimensional structure may compromise internal consistency reliability during application (91). Notably, Guo et al. (92) demonstrated limited applicability of Item F (“use of sleep medication”) in Chinese college populations. This limitation was reflected in our findings, where only 16 participants (3%) reported using sleep-aiding medications. To address these limitations, future studies could consider utilizing multivariate data collection methods that combine self-reporting with objective measures. For instance, physical activity levels could be quantified using accelerometers, phone addiction could be assessed through mobile app usage data, and sleep quality could be evaluated using sleep tracking devices. Additionally, incorporating observational data may further diminish the bias inherent in self-reporting.

Lastly, the cross-sectional nature of our study design limits our ability to establish causal relationships among variables. While our findings suggest that physical activity may reduce anxiety through decreased smartphone addiction, the possibility of reverse causality cannot be ruled out. For instance, individuals with lower anxiety levels might naturally engage in more physical activity and less smartphone use, or those with less smartphone dependency might have more time and motivation for physical activity. Future research should employ longitudinal designs and experimental interventions to better understand the temporal sequence and causal directions of these relationships.

## 6 Conclusion

This study revealed a more complex relationship between physical activity and anxiety than initially hypothesized. Our findings further revealed the relationship between physical activity and university students’ anxiety, and considered the mediating role of smartphone addiction between the two, as well as the moderating role of sleep quality in the relationship between mobile phone addiction and university students’ anxiety.

## Data Availability

The datasets presented in this article are not readily available because the data are not publicly available due to their containing information that could compromise the privacy of research participants. Requests to access the datasets should be directed to Qxr2001@outlook.com.

## Footnotes

^1^ www.gxu.edu.cn

^2^ https://www.wjx.cn

## References

[1] Gao H. Research on the construction of college students' mental health security system. J Healthc Eng. (2022) 2022:1–5. doi: 10.1155/2022/4001603, PMID: 35265299 PMC8901346

[2] Mao XL Chen HM. Investigation of contemporary college students' mental health status and construction of a risk prediction model. World J Psychiatry. (2023) 13:573–82. doi: 10.5498/wjp.v13.i8.573 37701543 PMC10494769

[3] Pan L Qiu W Hu Z Li J. Intolerance of uncertainty and internet addiction among college students in China post-pandemic era: the mediating role of future anxiety. Sci Rep. (2024) 14:20098. doi: 10.1038/s41598-024-70988-1, PMID: 39209922 PMC11362300

[4] Olthuis JV Watt MC Bailey K Hayden JA Stewart SH. Therapist-supported internet cognitive behavioural therapy for anxiety disorders in adults. Cochrane Database Syst Rev. (2016) 3:Cd011565. doi: 10.1002/14651858.CD011565.pub2 26968204 PMC7077612

[5] Spielberger CD Gorsuch RL Lushene RE. Manual for the state-trait anxiety inventory. Polo Alto, CA: Consulting Psychologists Press. (1970)

[6] Byrd-Bredbenner C Eck K Quick V. Psychometric properties of the generalized anxiety Disorder-7 and generalized anxiety disorder-Mini in United States university students. Front Psychol. (2020) 11:550533. doi: 10.3389/fpsyg.2020.550533, PMID: 33071867 PMC7541941

[7] Ströhle A Gensichen J Domschke K. The diagnosis and treatment of anxiety disorders. Dtsch Arztebl Int. (2018) 155:611–20. doi: 10.3238/arztebl.2018.0611, PMID: 30282583 PMC6206399

[8] Anderson E Shivakumar G. Effects of exercise and physical activity on anxiety. Front Psych. (2013) 4:27. doi: 10.3389/fpsyt.2013.00027, PMID: 23630504 PMC3632802

[9] Paluska SA Schwenk TL. Physical activity and mental health: current concepts. Sports Med. (2000) 29:167–80. doi: 10.2165/00007256-200029030-00003 10739267

[10] Wang M Chi S Wang X Wang T. Effects of tai Chi on anxiety and theta oscillation power in college students during the COVID-19 pandemic: a randomized controlled trial. PLoS One. (2024) 19:e0312804. doi: 10.1371/journal.pone.0312804, PMID: 39485780 PMC11530040

[11] Teychenne M Costigan SA Parker K. The association between sedentary behaviour and risk of anxiety: a systematic review. BMC Public Health. (2015) 15:513. doi: 10.1186/s12889-015-1843-x, PMID: 26088005 PMC4474345

[12] McMahon EM Corcoran P O'Regan G Keeley H Cannon M Carli V. Physical activity in European adolescents and associations with anxiety, depression and well-being. Eur Child Adolesc Psychiatry. (2017) 26:111–22. doi: 10.1007/s00787-016-0875-9, PMID: 27277894

[13] Gao Y Liu X Zhou Z Chao M Liu T. Data for developing computerized adaptive testing of problematic mobile phone use. Data Brief. (2024) 55:110746. doi: 10.1016/j.dib.2024.110746, PMID: 39183966 PMC11342194

[14] De-Sola Gutiérrez J Rodríguez de Fonseca F Rubio G. Cell-phone addiction: a review. Front Psychiatry. (2016) 7:175. doi: 10.3389/fpsyt.2016.00175, PMID: 27822187 PMC5076301

[15] Wang J Liu X Xu X Wang H Yang G. The effect of physical activity on sleep quality among Chinese college students: the chain mediating role of stress and smartphone addiction during the COVID-19 pandemic. Psychol Res Behav Manag. (2024) 17:2135–47. doi: 10.2147/prbm.S462794, PMID: 38826679 PMC11143986

[16] Višnjić A Veličković V Sokolović D Stanković M Mijatović K Stojanović M. Relationship between the manner of mobile phone use and depression, anxiety, and stress in university students. Int J Environ Res Public Health. (2018) 15:697. doi: 10.3390/ijerph15040697 29642471 PMC5923739

[17] Lepp A Barkley JE Sanders GJ Rebold M Gates P. The relationship between cell phone use, physical and sedentary activity, and cardiorespiratory fitness in a sample of U.S. college students. Int J Behav Nutr Phys Act. (2013) 10:79. doi: 10.1186/1479-5868-10-79 23800133 PMC3693866

[18] Lyu C Cao Z Jiao Z. Changes in Chinese college students' mobile phone addiction over recent decade: the perspective of cross-temporal meta-analysis. Heliyon. (2024) 10:e32327. doi: 10.1016/j.heliyon.2024.e32327 38947462 PMC11214489

[19] Zhao C Ding H Du M Yu Y Chen JH Wu AM. The vicious cycle between loneliness and problematic smartphone use among adolescents: a random intercept cross-lagged panel model. J Youth Adolesc. (2024) 53:1428–40. doi: 10.1007/s10964-024-01974-z, PMID: 38555341

[20] Cheng M Wang S Wang Y Zhang R Qin L. Physical activity reduces internet addiction among “post-00” college students: the mediating role of coping styles. Front Psychol. (2023) 13:1052510. doi: 10.3389/fpsyg.2022.1052510, PMID: 36846475 PMC9945914

[21] Du Z Zhang X. Analysis of the mediating effects of self-efficacy and self-control between physical activity and internet addiction among Chinese college students. Front Psychol. (2022) 13:1002830. doi: 10.3389/fpsyg.2022.1002830, PMID: 36211939 PMC9539857

[22] Zhuang J Mou Q Zheng T Gao F Zhong Y Lu Q. A serial mediation model of social media addiction and college students' academic engagement: the role of sleep quality and fatigue. BMC Psychiatry. (2023) 23:333. doi: 10.1186/s12888-023-04799-5, PMID: 37173670 PMC10176952

[23] Yao L Liang K Huang L Xiao J Zhou K Chen S. Longitudinal associations between healthy eating habits, resilience, insomnia, and internet addiction in Chinese college students: a cross-lagged panel analysis. Nutrients. (2024) 16:2470. doi: 10.3390/nu16152470 39125349 PMC11313817

[24] Höhn C Schmid SR Plamberger CP Bothe K Angerer M Gruber G. Preliminary results: the impact of smartphone use and Short-wavelength light during the evening on circadian rhythm, sleep and alertness. Clocks Sleep. (2021) 3:66–86. doi: 10.3390/clockssleep3010005, PMID: 33499010 PMC7838958

[25] Wang S Zhu C Dai H. Left-behind experience and mobile phone addiction among college students: a moderated mediation of social anxiety and sex. Heliyon. (2024) 10:e35452. doi: 10.1016/j.heliyon.2024.e35452, PMID: 39170196 PMC11336711

[26] Matar Boumosleh J Jaalouk D. Depression, anxiety, and smartphone addiction in university students- a cross sectional study. PLoS One. (2017) 12:e0182239. doi: 10.1371/journal.pone.0182239, PMID: 28777828 PMC5544206

[27] Meng S Zhang Y Tang L Zhang M Tang W Onyebuchi N. The effects of mobile phone addiction on bedtime procrastination in university students: the masking effect of physical activity and anxiety. BMC Psychol. (2024) 12:395. doi: 10.1186/s40359-024-01899-z, PMID: 39020420 PMC11253395

[28] Zhu W Liu J Lou H Mu F Li B. Influence of smartphone addiction on sleep quality of college students: the regulatory effect of physical exercise behavior. PLoS One. (2024) 19:e0307162. doi: 10.1371/journal.pone.0307162, PMID: 39058670 PMC11280214

[29] Billieux J Maurage P Lopez-Fernandez O Kuss DJ Griffiths MD. Can disordered Mobile phone use be considered a behavioral addiction? An update on current evidence and a comprehensive model for future research. Curr Addict Rep. (2015) 2:156–62. doi: 10.1007/s40429-015-0054-y, PMID: 40121490

[30] Li Y Li G Liu L Wu H. Correlations between mobile phone addiction and anxiety, depression, impulsivity, and poor sleep quality among college students: a systematic review and meta-analysis. J Behav Addict. (2020) 9:551–71. doi: 10.1556/2006.2020.00057, PMID: 32903205 PMC8943681

[31] Long J Liu TQ Liao YH Qi C He HY Chen SB. Prevalence and correlates of problematic smartphone use in a large random sample of Chinese undergraduates. BMC Psychiatry. (2016) 16:408. doi: 10.1186/s12888-016-1083-3, PMID: 27855666 PMC5114822

[32] Nikolic A Bukurov B Kocic I Vukovic M Ladjevic N Vrhovac M. Smartphone addiction, sleep quality, depression, anxiety, and stress among medical students. Front Public Health. (2023) 11:1252371. doi: 10.3389/fpubh.2023.1252371, PMID: 37744504 PMC10512032

[33] Mao Y Zhang N Liu J Zhu B He R Wang X. A systematic review of depression and anxiety in medical students in China. BMC Med Educ. (2019) 19:327. doi: 10.1186/s12909-019-1744-2, PMID: 31477124 PMC6721355

[34] Greszki R Meyer M Schoen H. The impact of speeding on data quality in nonprobability and freshly recruited probability-based online panels. Chichester: John Wiley & Sons, Ltd (2014).

[35] Bassett DR Jr. International physical activity questionnaire: 12-country reliability and validity. Med Sci Sports Exerc. (2003) 35:1396. doi: 10.1249/01.Mss.0000078923.96621.1d, PMID: 12900695

[36] Fan M Lyu J He P. Chinese guidelines for data processing and analysis concerning the international physical activity questionnaire. Chin J Epidemiol. (2014) 35:961–4. doi: 10.3760/cma.j.issn.0254-6450.2014.08.019, PMID: 25376692

[37] Craig CL Marshall AL Sjöström M Bauman AE Booth ML Ainsworth BE. International physical activity questionnaire: 12-country reliability and validity. Med Sci Sports Exerc. (2003) 35:1381–95. doi: 10.1249/01.Mss.0000078924.61453.Fb, PMID: 12900694

[38] Lynch L McCarron M McCallion P Burke E. An exploration into self-reported inactivity behaviours of adults with an intellectual disability using physical activity questionnaires. J Intellect Disabil Res. (2024) 68:1396–407. doi: 10.1111/jir.13184, PMID: 39229682

[39] Kwon M Kim DJ Cho H Yang S. The smartphone addiction scale: development and validation of a short version for adolescents. PLoS One. (2013) 8:e83558. doi: 10.1371/journal.pone.0083558, PMID: 24391787 PMC3877074

[40] Zhao H Rafik-Galea S Fitriana M Song TJ. Translation and psychometric evaluation of Smartphone Addiction Scale-Short Version (SAS-SV) among Chinese college students. PLoS One. (2022) 17:e0278092. doi: 10.1371/journal.pone.0278092, PMID: 36445890 PMC9707792

[41] Batista SA Ginani VC Stedefeldt E Nakano EY Botelho RBA. Reproducibility and validity of a self-administered food safety assessment tool on children and adolescent's risk perception, knowledge, and practices. Nutrients. (2023) 15:213. doi: 10.3390/nu15010213, PMID: 36615869 PMC9823607

[42] Buysse DJ Reynolds CF 3rd Monk TH Berman SR Kupfer DJ. The Pittsburgh sleep quality index: a new instrument for psychiatric practice and research. Psychiatry Res. (1989) 28:193–213. doi: 10.1016/0165-1781(89)90047-4, PMID: 2748771

[43] Zheng B Li M Wang KL Lv J. Analysis of the reliability and validity of the Chinese version of Pittsburgh sleep quality index among medical college students. J Pek Univ. (2016) 48:424–8. doi: 10.3969/j.issn.1671-167X.2016.03.009 27318902

[44] Zung WW. A rating instrument for anxiety disorders. Psychosomatics. (1971) 12:371–9. doi: 10.1016/s0033-3182(71)71479-0, PMID: 5172928

[45] Xu C Yifeng Y Weiming S Dan X Liang L Hayimu. Anxiety and its influencing factors among gay men infected with HIV /AIDS in Nanchang City. J Nanchang Univ. (2014) 54:84–6. doi: 10.13764/j.cnki.ncdm.2014.07.026

[46] Wen ZL Chang L Hau KT Liu HY. Testing and application of the mediating effects. Acta Psychol Sin. (2004) 15:227–32. doi: 10.1007/BF02911031, PMID: 40121490

[47] Preacher KJ Hayes AF. Asymptotic and resampling strategies for assessing and comparing indirect effects in multiple mediator models. Behav Res Methods. (2008) 40:879–91. doi: 10.3758/brm.40.3.879, PMID: 18697684

[48] Hooper D Mullen J Hooper D Coughlan J Mullen MR. Structural equation modeling: guidelines for determining model fit. Electr J Bus Res Methods. (2008) 6:53–60. doi: 10.0000/PMID35188134

[49] Hayes A. Introduction to mediation, moderation, and conditional process analysis. J Educ Meas. (2013) 51:335–7. doi: 10.1111/jedm.12050

[50] Chernick MR LaBudde RA. An introduction to bootstrap methods with applications to R2011. Hoboken: John Wiley & Sons, Inc (2011).

[51] Podsakoff PM MacKenzie SB Lee JY Podsakoff NP. Common method biases in behavioral research: a critical review of the literature and recommended remedies. J Appl Psychol. (2003) 88:879–903. doi: 10.1037/0021-9010.88.5.879, PMID: 14516251

[52] Bruno A Scimeca G Cava L Pandolfo G Zoccali RA Muscatello MRA. Prevalence of internet addiction in a sample of southern Italian high school students. Int J Ment Health Addict. (2014) 12:708–15. doi: 10.1007/s11469-014-9497-y PMC4221883 25401143

[53] Hayes AF Rockwood NJ. Regression-based statistical mediation and moderation analysis in clinical research: observations, recommendations, and implementation. Behav Res Ther. (2017) 98:39–57. doi: 10.1016/j.brat.2016.11.001, PMID: 27865431

[54] Schoemann AM Boulton AJ Short SD. Determining power and sample size for simple and complex mediation models. Soc Psychol Personal Sci. (2017) 8:379–86. doi: 10.1177/1948550617715068

[55] Wen Z Ye B. Mediation effect analysis: method and model development. Adv Psychol Sci. (2014) 22:731–45. doi: 10.3724/SP.J.1042.2014.00731, PMID: 37113526

[56] Chengming J Shu L. Mediation analysis and bootstrap application. Psychol Explor. (2015) 35:458–63. doi: 10.3969/j.issn.1003-5184.2015.05.013

[57] Zhao X Lynch JG Jr Chen Q. Reconsidering baron and Kenny: myths and truths about mediation analysis. J Consum Res. (2010) 37:197–206. doi: 10.1086/651257

[58] Liu Y Duan L Shen Q Ma Y Chen Y Xu L. The mediating effect of internet addiction and the moderating effect of physical activity on the relationship between alexithymia and depression. Sci Rep. (2024) 14:9781. doi: 10.1038/s41598-024-60326-w, PMID: 38684733 PMC11058241

[59] Xu Z Gao F Fa A Qu W Zhang Z. Statistical power analysis and sample size planning for moderated mediation models. Behav Res Methods. (2024) 56:6130–49. doi: 10.3758/s13428-024-02342-2, PMID: 38308148

[60] Lu X Wang Y Ma A. Study on the influence of test anxiety and psychological resilience game on the scores of physical education senior high school exams. J Nanj Sports Inst. (2021) 20:59–62. doi: 10.15877/j.cnki.nsin.2021.10.009

[61] Contreras DW Granquist MD Martin LA. Stress, sport anxiety, neuroticism, and coping in student-athletes: implications for patient mental health. J Athl Train. (2023) 58:733–9. doi: 10.4085/1062-6050-0527.22, PMID: 37248524 PMC11215728

[62] Putukian M. The psychological response to injury in student athletes: a narrative review with a focus on mental health. Br J Sports Med. (2016) 50:145–8. doi: 10.1136/bjsports-2015-095586, PMID: 26719498

[63] Rodriguez-Ayllon M Cadenas-Sánchez C Estévez-López F Muñoz NE Mora-Gonzalez J Migueles JH. Role of physical activity and sedentary behavior in the mental health of preschoolers, children and adolescents: a systematic review and meta-analysis. Sports Med. (2019) 49:1383–410. doi: 10.1007/s40279-019-01099-5 30993594

[64] Yang G Li Y Liu S Liu C Jia C Wang S. Physical activity influences the mobile phone addiction among Chinese undergraduates: the moderating effect of exercise type. J Behav Addict. (2021) 10:799–810. doi: 10.1556/2006.2021.00059, PMID: 34546969 PMC8997213

[65] Skinner BF. Science and human behavior. New York: Macmillan Publishers Limited. (1953).

[66] Bernstein EE Wolfe EC Huguenel BM Wilhelm S. Lessons and untapped potential of smartphone-based physical activity interventions for mental health: narrative review. JMIR Mhealth Uhealth. (2024) 12:e45860. doi: 10.2196/45860, PMID: 38488834 PMC10981024

[67] Dishman RK O'Connor PJ. Lessons in exercise neurobiology: the case of endorphins. Ment Health Phys Act. (2009) 2:4–9. doi: 10.1016/j.mhpa.2009.01.002

[68] Ghrouz AK Noohu MM Dilshad Manzar M Warren Spence D BaHammam AS Pandi-Perumal SR. Physical activity and sleep quality in relation to mental health among college students. Sleep Breath. (2019) 23:627–34. doi: 10.1007/s11325-019-01780-z 30685851

[69] Qin GY Han SS Zhang YS Ye YP Xu CY. Effect of physical exercise on negative emotions in Chinese university students: the mediating effect of self-efficacy. Heliyon. (2024) 10:e37194. doi: 10.1016/j.heliyon.2024.e37194, PMID: 39286123 PMC11402780

[70] Busch PA McCarthy S. Antecedents and consequences of problematic smartphone use: a systematic literature review of an emerging research area. Comput Hum Behav. (2021) 114:106414. doi: 10.1016/j.chb.2020.106414

[71] Wolfers LN Utz S. Social media use, stress, and coping. Curr Opin Psychol. (2022) 45:101305. doi: 10.1016/j.copsyc.2022.101305 35184027

[72] Wang R Ye B Wang P. Appearance comparison on social networking sites and body shame: the role of negative body talk and perceived sociocultural influences on body image. J Health Psychol. (2024) 30:224–37. doi: 10.1177/13591053241245100, PMID: 38600686

[73] Dittmar H Howard S. Thin-ideal internalization and social comparison tendency as moderators of media models' impact on women's body-focused anxiety. J Soc Clin Psychol. (2004) 23:768–91. doi: 10.1521/jscp.23.6.768.54799, PMID: 39183330

[74] Stice E Mazotti L Weibel D Agras WS. Dissonance prevention program decreases thin-ideal internalization, body dissatisfaction, dieting, negative affect, and bulimic symptoms: a preliminary experiment. Int J Eat Disord. (2000) 27:206–17. doi: 10.1002/(sici)1098-108x(200003)27:2<206:aid-eat9>3.0.co;2-d 10657894

[75] Fu T Wang J Xu S Yu J Sun G. Media internalized pressure and restrained eating behavior in college students: the multiple mediating effects of body esteem and social physique anxiety. Front Psychol. (2022) 13:887124. doi: 10.3389/fpsyg.2022.887124, PMID: 35783775 PMC9240773

[76] Shen X Wang C Chen C Wang Y Wang Z Zheng Y. Stress and internet addiction: mediated by anxiety and moderated by self-control. Psychol Res Behav Manag. (2023) 16:1975–86. doi: 10.2147/prbm.S411412, PMID: 37284553 PMC10239643

[77] Ouyang Y Luo J Teng J Zhang T Wang K Li J. Research on the influence of media internalized pressure on college students' sports participation-chained intermediary analysis of social physique anxiety and weight control self-efficacy. Front Psychol. (2021) 12:654690. doi: 10.3389/fpsyg.2021.654690, PMID: 34054659 PMC8149783

[78] King ALS Pádua MK Gonçalves LL de Souza S Martins A Nardi AE. Smartphone use by health professionals: a review. Digit Health. (2020) 6:2055207620966860. doi: 10.1177/2055207620966860, PMID: 33294206 PMC7708699

[79] Zhao X Hu T Qiao G Li C Wu M Yang F. Psychometric properties of the smartphone distraction scale in Chinese college students: validity, reliability and influencing factors. Front Psych. (2022) 13:859640. doi: 10.3389/fpsyt.2022.859640, PMID: 35782432 PMC9243416

[80] Throuvala MA Griffiths MD Rennoldson M Kuss DJ. Mind over matter: testing the efficacy of an online randomized controlled trial to reduce distraction from smartphone use. Int J Environ Res Public Health. (2020) 17:4842. doi: 10.3390/ijerph17134842 32635650 PMC7369880

[81] Demirci K Akgönül M Akpinar A. Relationship of smartphone use severity with sleep quality, depression, and anxiety in university students. J Behav Addict. (2015) 4:85–92. doi: 10.1556/2006.4.2015.010, PMID: 26132913 PMC4500888

[82] Gong L Liu Q. Mobile phone addiction and sleep quality: the mediating role of anxiety and the moderating role of emotion regulation. Behav Sci (Basel). (2023) 13:250. doi: 10.3390/bs13030250 36975275 PMC10045665

[83] Chue AE Gunthert KC Kim RW Alfano CA Ruggiero AR. The role of sleep in adolescents' daily stress recovery: negative affect spillover and positive affect bounce-back effects. J Adolesc. (2018) 66:101–11. doi: 10.1016/j.adolescence.2018.05.006, PMID: 29842996

[84] Kim SY Han S Park EJ Yoo HJ Park D Suh S. The relationship between smartphone overuse and sleep in younger children: a prospective cohort study. J Clin Sleep Med. (2020) 16:1133–9. doi: 10.5664/jcsm.8446, PMID: 32248898 PMC7954067

[85] Lam LT. Risk factors of internet addiction and the health effect of internet addiction on adolescents: a systematic review of longitudinal and prospective studies. Curr Psychiatry Rep. (2014) 16:508. doi: 10.1007/s11920-014-0508-2, PMID: 25212714

[86] Tsai PS Wang SY Wang MY Su CT Yang TT Huang CJ. Psychometric evaluation of the Chinese version of the Pittsburgh sleep quality index (CPSQI) in primary insomnia and control subjects. Qual Life Res. (2005) 14:1943–52. doi: 10.1007/s11136-005-4346-x, PMID: 16155782

[87] Yang J Fu X Liao X Li Y. Association of problematic smartphone use with poor sleep quality, depression, and anxiety: a systematic review and meta-analysis. Psychiatry Res. (2020) 284:112686. doi: 10.1016/j.psychres.2019.112686, PMID: 31757638

[88] Chellappa SL Aeschbach D. Sleep and anxiety: from mechanisms to interventions. Sleep Med Rev. (2022) 61:101583. doi: 10.1016/j.smrv.2021.101583, PMID: 34979437

[89] Hall BJ Xiong P Guo X Sou EKL Chou UI Shen Z. An evaluation of a low intensity mHealth enhanced mindfulness intervention for Chinese university students: a randomized controlled trial. Psychiatry Res. (2018) 270:394–403. doi: 10.1016/j.psychres.2018.09.060, PMID: 30300870

[90] Exelmans L Van den Bulck J. Bedtime, shuteye time and electronic media: sleep displacement is a two-step process. J Sleep Res. (2017) 26:364–70. doi: 10.1111/jsr.12510, PMID: 28271575

[91] Nicassio PM Ormseth SR Custodio MK Olmstead R Weisman MH Irwin MR. Confirmatory factor analysis of the Pittsburgh sleep quality index in rheumatoid arthritis patients. Behav Sleep Med. (2014) 12:1–12. doi: 10.1080/15402002.2012.720315, PMID: 23390921 PMC4285368

[92] Guo S Sun W Liu C Wu S. Structural validity of the Pittsburgh sleep quality index in Chinese undergraduate students. Front Psychol. (2016) 7:1126. doi: 10.3389/fpsyg.2016.01126, PMID: 27551270 PMC4976124

